# Light sources and detectors: applications in nanoscale imaging, sensing, and actuation

**DOI:** 10.1117/1.JBO.31.6.064301

**Published:** 2026-05-25

**Authors:** Sebastian Wachsmann-Hogiu, Monica Focşan, Dror Fixler

**Affiliations:** a McGill University, Department of Biomedical Engineering, Montreal, Québec, Canada; b Babes-Bolyai University, Interdisciplinary Research Institute in Bio-Nano-Sciences, Nanobiophotonics and Laser Microspectroscopy Center, Cluj-Napoca, Romania; c Babes-Bolyai University, Faculty of Physics, Biomolecular Physics Department, Cluj-Napoca, Romania; d Tel Aviv University, Faculty of Engineering, School of Biomedical Engineering, Jan Koum Center for Nanoscience and Nanotechnology, Light Matter Interaction Center, Tel Aviv, Israel

## Abstract

The editorial provides an overview of the JBO Special Section Nanoscale Imaging, Sensing, and Actuation and reflects on the future of nanoscale biophotonics.

Advances in biomedical optics are increasingly driven by the co-evolution of optical sources, detector technologies, nanostructured interfaces, and quantitative measurement strategies. What once appeared as separate development tracks—novel photonic materials, improved sensing platforms, polarization-aware detection, and computationally informed retrieval of tissue parameters—are now converging into integrated systems for imaging, sensing, and actuation. The articles collected in the JBO special section “Nanoscale Imaging, Sensing, and Actuation” reflect that convergence particularly well. Taken together, they show how progress at the nanoscale can influence performance at the system level, and how innovations in measurement geometry, light–matter interaction, and signal interpretation can expand the practical reach of biophotonics.

A recurring theme throughout this collection is that the future of nanoscale biophotonics will depend not only on making structures smaller or detectors more sensitive, but also on designing complete optical frameworks that are robust in realistic biological environments. This includes the ability to control optical fields at interfaces, to suppress or exploit polarization effects, to understand signal transduction at nanoparticle–biomolecule surfaces, and to recover quantitative tissue parameters without excessive dependence on external calibration. In that sense, this special section captures an important shift in the field: from isolated demonstrations of optical capability toward functionally meaningful and application-aware biophotonic platforms.

The special section opens with the roadmap paper, “Roadmap for light interaction with biophotonic surfaces and their diverse applications.”^1^ This contribution is especially valuable because it provides a broad conceptual and technological view of how biophotonic surfaces can guide light in ways that are useful for biomedical imaging, sensing, and actuation. Roadmap articles play a distinctive role in interdisciplinary fields: they do not merely summarize progress, but define the scientific architecture of a research area, identify emerging opportunities, and help align communities that may otherwise remain fragmented across materials science, optics, bioengineering, and device development. In that respect, this paper serves as both an overview and a forward-looking framework for the broader themes represented in the special section. This paper is a result of PhoBioS COST Action that has led to many interesting new directions in biophotonics.^2^

The second paper, “Polarizer-assisted pupillometry through closed eyelids, overcoming pupil position dependence,”^3^ which features on the issue cover, addresses a highly practical challenge in biomedical optical measurement. By introducing polarization-assisted SWIR pupillometry and explicitly confronting the issue of pupil-position dependence, the study demonstrates how careful optical design can improve robustness under difficult physiological and geometrical conditions. Its importance extends beyond the specific application of pupillometry. More broadly, it exemplifies how polarization can be used not only as a contrast mechanism, but also as a stabilizing design variable that makes noninvasive measurements less vulnerable to anatomical variability and uncontrolled optical paths. This kind of work is essential if biophotonic methods are to move reliably from laboratory settings into demanding real-world use.

At the nanoscale sensing interface, “DNA-hybridization on gold nanospheres: a dual-fluorescence investigation of surface loading and strand length effects”^4^ provides an instructive example of how nanoparticle-enabled optical assays are becoming increasingly quantitative. Rather than stopping at signal demonstration, the authors examine how surface loading and strand length influence fluorescence response and hybridization behavior. This is scientifically important because biosensing performance in such systems depends critically on nanoscale organization: fluorophore–metal separation, local quenching environments, steric effects, and surface density all shape the observable optical signal. By probing these dependencies, the paper contributes to a more mechanistic understanding of plasmonic nanosensors and supports the broader movement toward rational design rules for optical biosensing platforms.

The fourth paper, “Endoscopic iso-pathlength self-calibration for direction-resolved retrieval of tissue optical properties,”^5^ represents a particularly significant advance in quantitative tissue optics. The central challenge in diffuse and scattering-based measurements has long been the reliable extraction of tissue optical properties under realistic conditions, where absorption, scattering, geometry, and instrumentation are tightly coupled. By bringing the iso-pathlength self-calibration concept into an endoscopic implementation and coupling it with direction-resolved retrieval, this work addresses that challenge directly. It is notable not only for its methodological sophistication, but also for its translational relevance: self-calibrated approaches are especially attractive in endoscopic and clinical contexts, where external calibration can be cumbersome or unstable. This study therefore stands at the intersection of theoretical optical insight, instrumentation design, and practical biomedical utility.

The endoscopic iso-pathlength paper^5^ also fits into a broader scientific trajectory in quantitative near-infrared and tissue-optics research. Earlier studies helped establish the self-calibration and iso-pathlength framework through theory,^6^ simulation, benchtop validation, *in vivo* measurements, single-wavelength absorption retrieval, and analysis of the influence of detector size and positioning on the apparent self-calibration point. Mentioning that lineage is appropriate here, because it illustrates how concepts first developed in controlled optical studies can mature into more application-ready biomedical implementations.^7^

In this context, it is also worth noting the recent JBO Perspective by Hughes, Upadhya, and Dholakia.^8^ Although this article, “Superconducting nanowire single-photon detectors for enhanced biomedical imaging,” was published outside the present section, it resonates strongly with the special section’s central themes. The article highlights how detector innovation—specifically the emergence of superconducting nanowire single-photon detectors—can transform photon-limited biomedical imaging through exceptional sensitivity, picosecond timing resolution, and broad spectral responsiveness. Its inclusion in this wrap-up discussion is particularly apt because it reminds us that the future of nanoscale imaging and sensing depends not only on better probes, surfaces, and retrieval models, but also on detectors capable of observing weaker, faster, and more information-rich optical signals.

Taken as a whole, these collected works point toward several larger directions for the field. First, optical performance in biomedical systems is increasingly achieved through integration, not isolation: sources, surfaces, detectors, and algorithms must be designed in concert. Second, nanoscale platforms are becoming more quantitatively interpretable, allowing signal changes to be linked to physical structure and local optical environment rather than treated as empirical outputs alone. Third, there is a clear shift toward robustness—robustness to geometry, tissue variability, environmental perturbation, and deployment constraints. This trend is essential if nanoscale optical technologies are to mature into clinically and biologically impactful tools. We continue to organize the related BiOS conference at SPIE Photonics West and look forward to the broader participation of the community.^9^

We hope this JBO special section will be useful both as a snapshot of current activity and as a marker of the direction in which the field is moving. The contributions gathered here demonstrate that nanoscale imaging, sensing, and actuation are no longer peripheral themes in biomedical optics; they are increasingly central to how new optical technologies are conceived, validated, and translated. We are grateful to all authors for their outstanding contributions, to the reviewers for their thoughtful and constructive assessments, and to the editorial team of the *Journal of Biomedical Optics* for their support in bringing this special section into publication.
